# Case report: Emapalumab for active disease control prior to hematopoietic stem cell transplantation in refractory systemic juvenile idiopathic arthritis complicated by macrophage activation syndrome

**DOI:** 10.3389/fped.2023.1123104

**Published:** 2023-02-20

**Authors:** Deepak Chellapandian, Diana Milojevic

**Affiliations:** ^1^ Center for Cell and Gene Therapy for Nonmalignant Conditions, Blood and Marrow Transplant Program, Johns Hopkins All Children’s Hospital, St Petersburg, FL, United States; ^2^ Division of Pediatric Rheumatology, Johns Hopkins All Children’s Hospital, St Petersburg, FL, United States

**Keywords:** case report, systemic juvenile idiopathic arthritis (sJIA), macrophage activation syndrome (MAS), emapalumab, allogeneic hematopoietic stem cell transplantation

## Abstract

**Introduction:**

Macrophage activation syndrome (MAS), a secondary form of hemophagocytic lymphohistiocytosis, is a serious life-threatening complication associated with systemic juvenile idiopathic arthritis (sJIA). MAS is characterized by fever, hepatosplenomegaly, liver dysfunction, cytopenias, coagulation abnormalities, and hyperferritinemia and may progress to multiple organ failure and death. Overproduction of interferon-gamma is a major driver of hyperinflammation in murine models of MAS and primary hemophagocytic lymphohistiocytosis. A subset of patients with sJIA may develop progressive interstitial lung disease, which is often difficult to manage. Allogeneic hematopoietic stem cell transplantation (allo-HSCT) can potentially be a curative immunomodulatory strategy for patients with sJIA refractory to conventional therapy and/or complicated by MAS. The use of emapalumab (anti-interferon gamma antibody) for the active control of MAS in refractory cases of sJIA and associated lung disease has not been reported. Herein we report a patient with refractory sJIA complicated by recurrent MAS and lung disease that was managed with emapalumab and ultimately followed by an allo-HSCT, which resulted in permanent correction of the underlying immune dysregulation and improvement of lung disease.

**Case Report:**

We present a 4-year-old girl with sJIA complicated by recurrent MAS and progressive interstitial lung disease. She developed a progressively worsening disease that was refractory to glucocorticoids, anakinra, methotrexate, tocilizumab, and canakinumab. She had a chronic elevation of serum inflammatory markers, notably soluble interleukin-18, and CXC chemokine ligand 9 (CXCL9). Emapalumab, initiated at 6 mg/kg (1 dose) and continued at 3 mg/kg twice weekly for a total of 4 weeks, resulted in MAS remission along with normalization of inflammatory markers. The patient received a matched sibling donor allo-HSCT after a reduced-intensity conditioning regimen with fludarabine/melphalan/thiotepa and alemtuzumab, along with tacrolimus and mycophenolate mofetil for graft-vs.-host disease prophylaxis. At 20 months following her transplant, she has maintained a full donor engraftment with complete donor-derived immune reconstitution. She had complete resolution of sJIA symptoms including marked improvement in her lung disease along with normalization of serum interleukin-18 and CXCL9 levels.

**Conclusion:**

The use of emapalumab followed by allo-HSCT could help achieve a complete response in refractory cases of sJIA complicated by MAS who have failed standard treatment.

## Introduction

Systemic juvenile idiopathic arthritis (sJIA) is a severe autoinflammatory disorder in early childhood that accounts for 10%–20% of juvenile idiopathic arthritis in the Caucasian populations of Northern America and Europe (1, 2). The clinical presentation of sJIA is characterized by prominent systemic features such as spiking fevers, evanescent erythematous rashes, generalized lymphadenopathy, hepatosplenomegaly, and serositis (3). Macrophage activation syndrome (MAS), a secondary form of hemophagocytic lymphohistiocytosis (HLH), is a serious and potentially fatal complication seen in up to 10% of patients with sJIA resulting from uncontrolled activation of the immune system and overproduction of cytokines (1, 4, 5). The increasing use of targeted biologic agents against interleukin (IL)-1 (anakinra, canakinumab) and IL-6 (tocilizumab) has dramatically improved the long-term outcomes of the disease and reduced the need for glucocorticoids (2). Despite the improved control of sJIA, the targeted biological agents do not protect against the development of MAS, and the rates of mortality, as high as 20%–30%, remain similar (6, 7).

In recent years, children with sJIA have been increasingly found to have more severe and life-threatening lung disease (LD) presenting as pulmonary hypertension, interstitial LD, and pulmonary alveolar proteinosis (5). The immunopathological basis of LD is unclear, but it has been postulated that these complications could be related to uncontrolled sJIA activity and/or related to the increasing use of cytokine-directed biologics in these patients (2, 8).

Allogeneic (allo-) hematopoietic stem cell transplantation (HSCT) is an increasingly recognized curative treatment for various forms of monogenic immune dysregulation disorders (9). The allo-HSCT has a potential advantage in this context, with the goal of replacing the defective immune system, thereby achieving lifelong immunosuppression-free disease remission (10). Achieving clinical and biological disease control prior to allo-HSCT is critical and could be challenging with the available biological agents (9). Neutralization of interferon-gamma (IFNγ) using emapalumab, an IFN*γ*-blocking antibody, is an effective strategy to achieve disease control in primary HLH prior to allo-HSCT (9). Its utility in MAS in sJIA patients and adult-onset Still's disease is currently being investigated in a prospective clinical trial (NCT05001737). Herein we report a case of refractory sJIA with MAS and associated LD that was effectively managed using emapalumab and further proceeded to an allo-HSCT to achieve permanent disease control with marked improvement in LD.

## Case description

**Figure 1:**
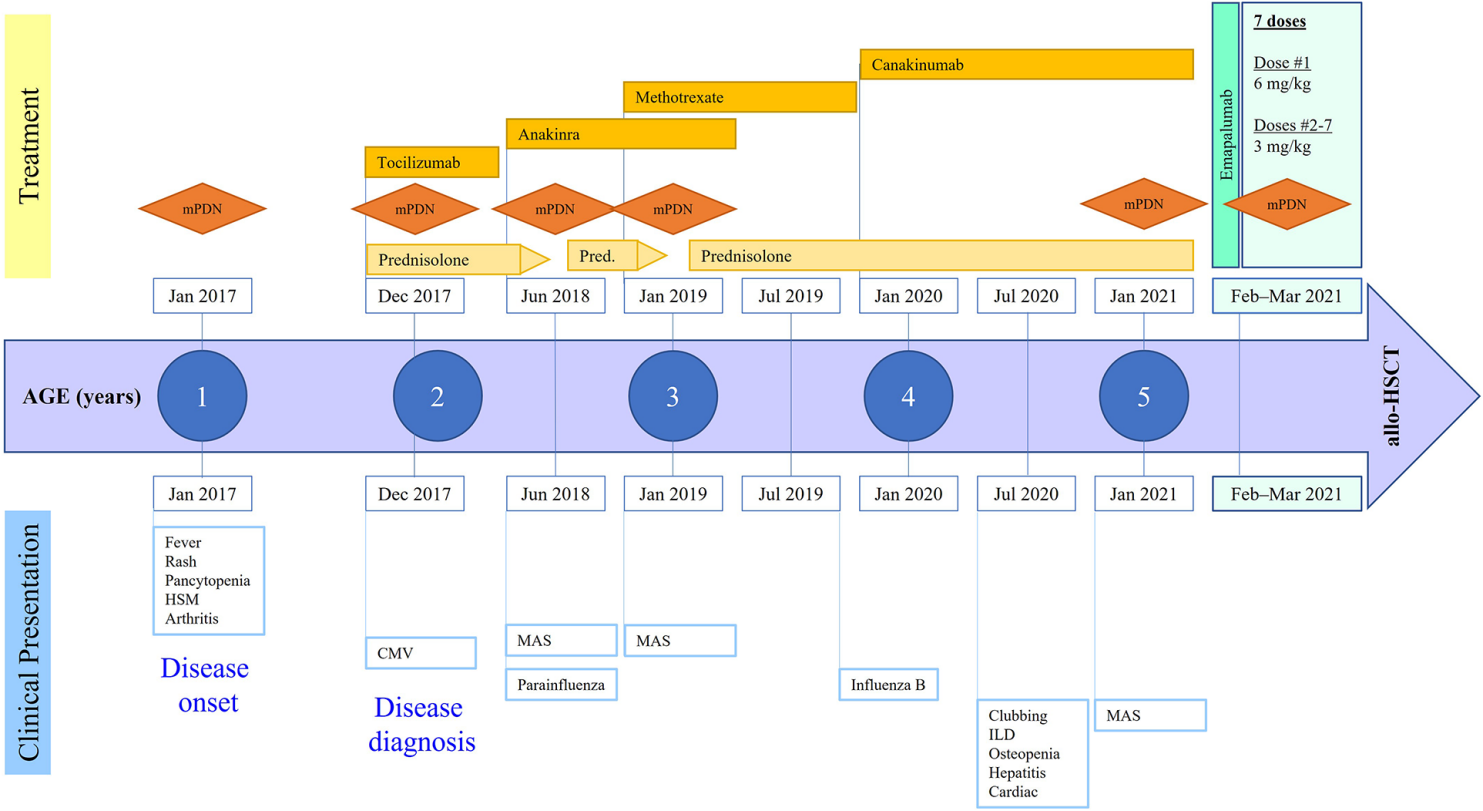
Disease course and treatment over time. mPDN, methylprednisolone; HSM, hepatosplenomegaly; CMV, cytomegalovirus; ILD, interstitial lung disease; MAS, macrophage activation syndrome; allo-HSCT, allogeneic hematopoietic stem cell transplantation.

This female patient first presented at 21 months of age with a high-grade fever up to 40°C, evanescent macular rash, arthritis of bilateral knees, and generalized lymphadenopathy, thereby meeting the criteria for sJIA according to the Pediatric Rheumatology International Trials Organization international consensus (11). She was born full-term *via* normal vaginal delivery to non-consanguineous parents. A full timeline presenting historical findings and treatment over time is shown in Figure 1. Prior to this episode, the patient had presented to her pediatrician multiple times with recurrent fever and rash that were mostly treated as viral illnesses. At presentation, she was noted to have prominent hepatosplenomegaly, pancytopenia (white blood cell count 4,100/ml, hemoglobin 9.8 g/dl, platelet count 98,000/ml), elevated liver enzymes (aspartate transaminase 78 IU/L, alanine transaminase 110 IU/L), hypertriglyceridemia (270 mg/dl), and elevated inflammatory markers including C-reactive protein (18.6 mg/dl), erythrocyte sedimentation rate (70 mm/h), and ferritin (>10,000 ng/ml). She was diagnosed with MAS complicating underlying sJIA. Her bone marrow examination showed mature trilineage hematopoiesis without any evidence of hemophagocytosis. The gene sequencing panel did not reveal any abnormalities for primary immune deficiency disorders including primary HLH. She also had detectable copies of cytomegalovirus (500 IU/ml) in the plasma during this time. She was treated with a pulse dose of intravenous methylprednisolone (mPDN) at 30 mg/kg along with non-steroidal anti-inflammatory drugs. She demonstrated resolution of fever and rash along with improvement of her inflammatory markers, after which she was switched to systemic oral prednisolone at 2 mg/kg/day. During this admission she also received her first dose of tocilizumab (IL-6 receptor antagonist) which she tolerated; however, she developed severe infusion reactions to subsequent doses that necessitated cessation of further therapy. Daily anakinra (IL-1 receptor antagonist) was initiated at 2 mg/kg as a disease-modifying agent. She also received an intra-articular injection of corticosteroids to her knees for the treatment of arthritis.

She was gradually weaned off oral steroids but had a relapse of MAS flare at 30 months of age following a parainfluenza 3 viral infection. She was hospitalized, treated with pulse mPDN, and then continued on oral steroids with an escalation of the anakinra dose to 3 mg/kg/day. In another attempt to wean her off oral steroids, she developed relapse of MAS symptoms at 36 months of age, presenting as recurrent fever and worsening hepatosplenomegaly. She demonstrated significant elevation of IL-18 (353,667 pg/ml, normal range: 89–540), soluble IL-2 receptor (135,495 U/ml, normal <2,400), and soluble CD163 (2,022 ng/ml, normal range 399–1,988). She required a pulse dose of mPDN this time followed by an increase in her daily prednisolone dose to 2 mg/kg/day. The dose of anakinra was further increased to 4 mg/kg/day. Given the recurrent nature of the disease and to help taper her off corticosteroids, she was started on weekly methotrexate (10 mg, subcutaneous) as an additional disease-modifying agent. Subsequently, she developed several viral illnesses including influenza B, coxsackie, and rhinovirus, all of which resulted in worsening of sJIA inflammation and escalation of immunosuppression therapy. She developed cushingoid features from chronic steroid exposure including excessive weight gain, bilateral cataracts, and orthopedic complications such as subluxation of the hip joint and multiple vertebral fractures without spinal cord compression. Although this patient had recurrent episodes of MAS, treatment with cyclosporine was not considered because of her worsening hypertension and chronic steroid exposure.

## Additional clinical findings and diagnostic assessment

**Figure 2:**
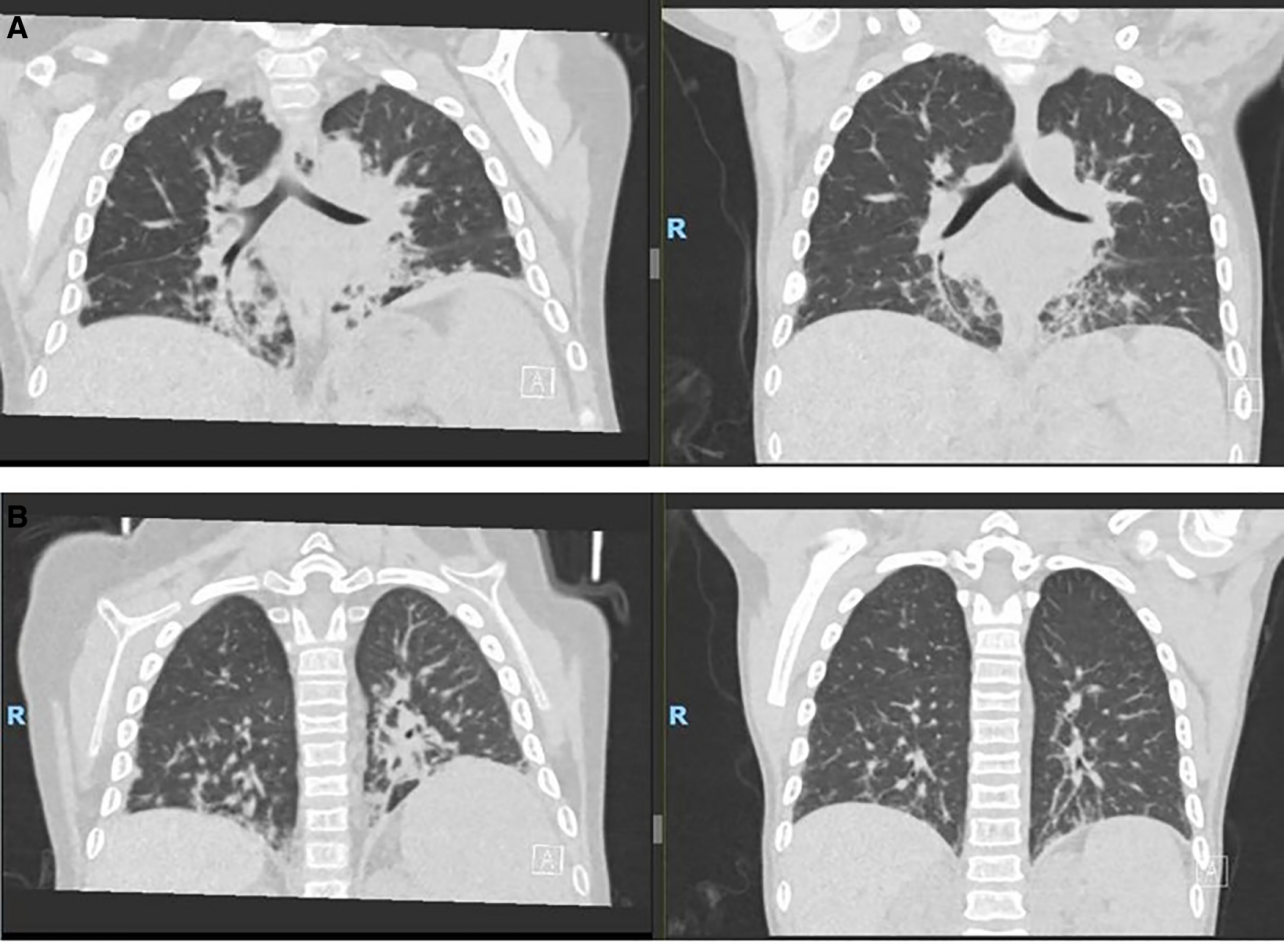
Ct scans. (**A**) Pre–allo-HSCT. (**B**) 9 months post–allo-HSCT. CT, computed tomography; allo-HSCT, allogeneic hematopoietic stem cell transplantation.

The patient was admitted to an intensive care unit at 4.5 years of age with pneumonia and acute respiratory distress syndrome that required intubation and ventilatory support. During further evaluation of her lungs for ongoing respiratory support and with the development of new-onset clubbing, a high-resolution computed tomography (CT) scan showed interstitial LD with bilateral multilobar parenchymal changes with lower lobe predominance (Figure 2A). A wedge biopsy of her lung demonstrated a chronic active interstitial fibroinflammatory process with foamy macrophages, cholesterol clefts, and chronic accumulation of lipoprotein material, all consistent with chronic LD associated with autoimmune disease. At the time, the patient was too young to undergo pulmonary function testing; however, she did not require oxygen and maintained an oxygen saturation >95% when breathing room air. Due to possible implications of the lung findings from the usage of anakinra and methotrexate, these agents were discontinued, and the patient was switched to canakinumab (an IL-1β blocker) at a dose of 4 mg/kg every 4 weeks. We note that at the time of interstitial LD diagnosis, her absolute eosinophil count was 100 per mm^3^. High resolution HLA typing did not identify an immunogenetic predisposition in this patient including HLA-DRB1*15 allele.

## Therapeutic intervention using emapalumab before allo-HSCT

The patient was referred to the bone marrow transplantation team due to exhaustion of other medical management and the development of worsening treatment-related toxicities. At that time, her IL-18 level was 299,320 pg/ml and her CXC chemokine ligand 9 (CXCL9) level was 17,165 pg/ml (normal <121). Her younger female sibling was identified as a human leukocyte antigen (HLA)–matched donor. After obtaining informed consent from the family for the procedure, transplant preparation and workup were initiated. During the process, the patient, unfortunately, developed an episode of MAS that delayed her from proceeding to transplant. She presented with a high-grade fever, worsening cytopenia, hypertriglyceridemia, and elevated inflammatory markers (Table 1). She required a pulse dose of mPDN (30 mg/kg). She had an acute elevation in CXCL9 levels and given the role of IFN*γ* as the pivotal cytokine in mediating MAS flares, emapalumab, which was acquired commercially for this off-label use, was initiated. Following the dosing used in a pilot study of emapalumab in patients with sJIA, she received a loading dose of emapalumab at 6 mg/kg/day followed by twice-weekly doses of 3 mg/kg for six doses (12). Two weeks after initiating emapalumab, the patient developed fever with acute elevation of CRP levels while experiencing ongoing cytopenia and hyperferritinemia and receiving maintenance corticosteroids. This led us to suspect an impending disease flare (sJIA vs. incomplete MAS). Thus, she received an additional pulse dose of mPDN (30 mg/kg). The patient was maintained on oral prednisolone (between 0.5 and 1 mg/kg/d) for the duration of her emapalumab treatment. She had a complete resolution of her disease flare symptoms upon completion of the planned seven doses of emapalumab. Correspondingly, her inflammatory markers ferritin (2,144 ng/ml) and CXCL9 (254 pg/ml) were substantially improved (Table 1).

**Table 1:** Laboratory parameters during emapalumab treatment.

	Emapalumab treatment			
	Day 0 (initiation)	Week 1	Week 2	Week 3
Hemoglobin (g/dl)	7.4	10.5	9.4	10.5
White blood cells (1,000/mm^3^)	1.85	1.14	3.9	14
Neutrophils (1,000/mm^3^)	1.41	0.6	0.6	11.2
Absolute lymphocyte count (1,000/mm^3^)	0.35	–	–	1.2
Platelets (1,000/mm^3^)	49	86	178	216
Ferritin (ng/ml)	>40,000	17,275	9,237	2,144
C-reactive protein (mg/dl)	22.46	0.28	15.7	2.43
CXCL-9 (pg/ml)	17,165	490	274	254
IL-18 (pg/ml)	471,860	–	–	210,062
Triglycerides (mg/dl)	472	299	–	228
Soluble IL-2R (U/ml)	2,556	–	–	1,426

CXCL, CXC chemokine ligand; IL, interleukin.

## Follow-up and outcomes following allo-HSCT

The patient underwent an allo-HSCT immediately after achieving remission of MAS using reduced-intensity conditioning with fludarabine (150 mg/m^2^, days –7 to –3), melphalan (140 mg/m^2^, day –2), and thiotepa (8 mg/kg, day –3), along with alemtuzumab (1 mg/kg, days –14 to –10) for serotherapy. She received an unmanipulated ABO-compatible bone marrow graft from her HLA-identical sibling. She developed another episode of MAS immediately following the administration of alemtuzumab that manifested as fever, rising ferritin and C-reactive protein, and acute hemolysis, all of which resolved with a pulse dose of mPDN. The total CD34 content of the graft was 6.47 × 10^6^ per kilogram of the recipient's weight. Tacrolimus and mycophenolate mofetil were used for graft-vs.-host disease prophylaxis. She engrafted neutrophils on day +15 and platelets on day +40, following the stem cell infusion. Her post-transplant course was complicated by moderate to severe mucositis requiring parenteral nutrition, gastroenteritis, prolonged refractory thrombocytopenia, engraftment syndrome, fluid overload with hypertension, transaminitis, and hypogammaglobinemia.

**Figure 3:**
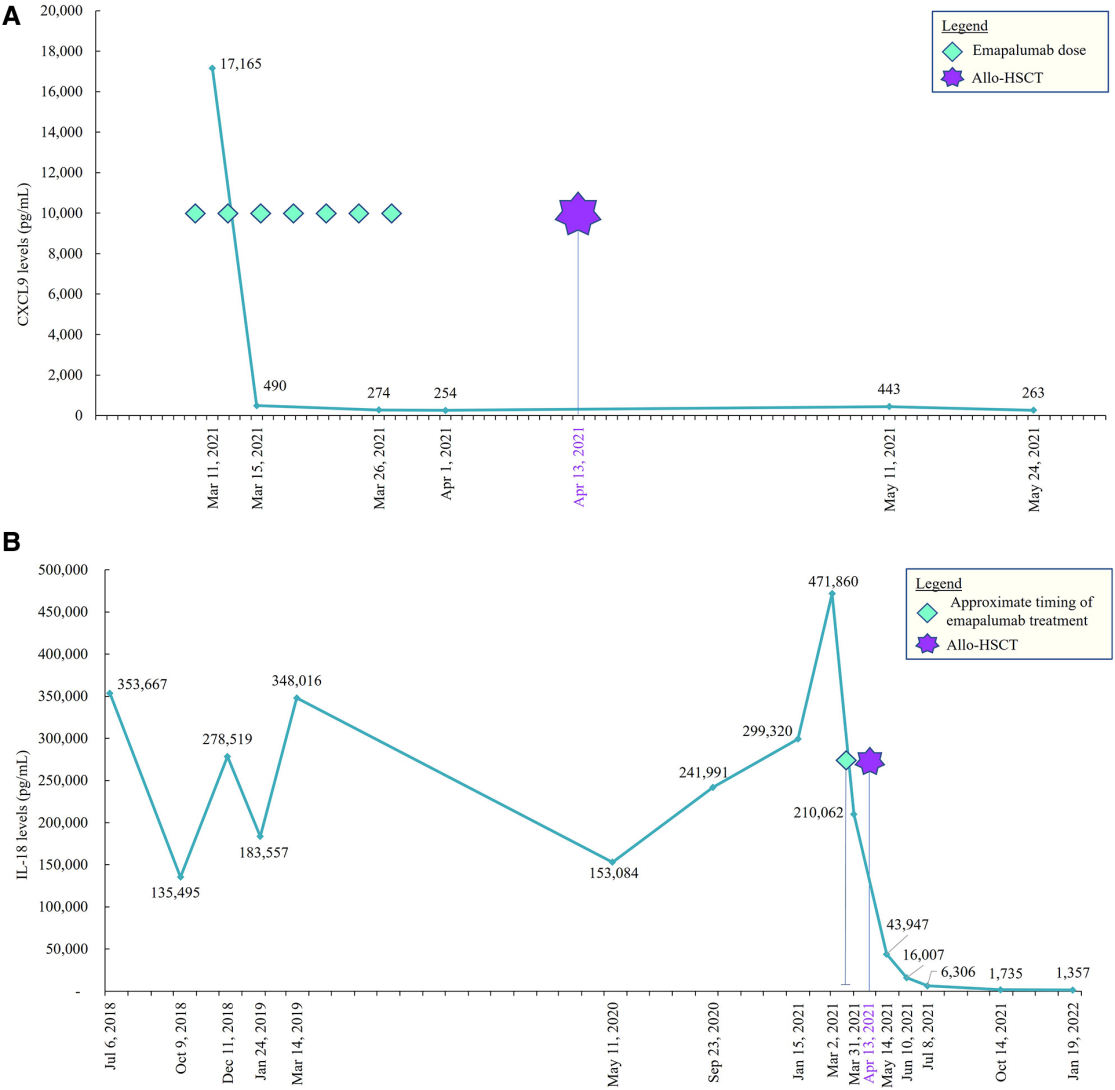
(**A**) CXCL9 pre– and post–allo-HSCT. CXCL9, CXC chemokine ligand 9; allo-HSCT, allogeneic hematopoietic stem cell transplantation. (**B**) IL-18 pre– and post–allo-HSCT. IL, interleukin; allo-HSCT, allogeneic hematopoietic stem cell transplantation.

She reactivated cytomegalovirus in her plasma without end-organ disease and was managed with foscarnet and valganciclovir therapy. She had developed new onset cardiac dysfunction associated with left ventricular outlet tract obstruction during transplant, which required aggressive diuresis, angiotensin-converting enzyme inhibitors, and beta-blockers. All her cardiac manifestations resolved completely by approximately 3 months following the transplant. She had no signs of acute or chronic graft-vs.-host disease, and all her immunosuppressive medications were tapered and discontinued by around 6 months. She attained full donor chimera in all her cell lineages at day +30 and maintained stability until the last follow-up post-transplant. She did not have any recurrence of MAS episodes after the transplant, and her inflammatory markers down-trended with near normalization by 1 year after the transplant (Figures 3A, B). She did not have any recurrence of pulmonary symptoms, and her post-transplant lung CT scan at 9 months revealed significant improvement in multifocal airspace opacities that were noted prior to transplant (Figure 2B).

At 20 months follow-up after HSCT, the patient maintained full donor chimerism, without signs of graft vs. host disease or organ dysfunction. Levels of inflammatory markers, including IL18, were normalized. She was not receiving immunoglobulin supplementation, was fully revaccinated, and returned to in-person school. The patient had ongoing osteopenia from steroid exposure and was on vitamin D supplementation.

## Discussion

This case study demonstrates that neutralization of IFNγ using emapalumab can be an effective strategy to control MAS in refractory cases of sJIA. Allo-HSCT using a reduced intensity conditioning regimen could be a feasible immune modulatory approach in severe sJIA that is refractory to conventional therapy and/or complicated by recurrent MAS. Our patient received emapalumab along with pulse doses of corticosteroids, which helped control MAS symptoms and demonstrated a rapid reduction of inflammatory markers prior to allo-HSCT. Post-transplant she demonstrated significant improvement in her LD with continued normalization of inflammatory markers, all consistent with the correction of underlying immune dysregulation.

Genetic predisposition to sJIA is mainly due to HLA class II molecules (HLA-DRB1, HLA-DPB1), although HLA class I molecules and non-HLA genes have also been implicated (13). Associations with MHC Class II have been described for sJIA, these include HLA DRB1:04:05 and DQB1:04:01 (14). Drug reactions such as eosinophilia and reactions with systemic symptoms have been reported in patients taking IL-1/IL-6 inhibitors; these reactions are known to be associated with HLADRB1*15 haplotypes (15). High resolution HLA typing in our patient did not identify an immunogenetic predisposition.

Targeted biological agents such as anakinra, canakinumab, and tocilizumab have been shown to maintain control of sJIA and reduce the need for glucocorticoids (2); however, available biological agents have not been assessed to understand the impact on the rate and clinical presentation of MAS development in patients with sJIA. Acute pulmonary dysfunction is commonly seen in sJIA, particularly during acute MAS, and chronic parenchymal LD has rarely been seen in sJIA in North America prior to 2013 (7). Systemic JIA-associated LD has increasingly been recognized in the past decade, especially in patients treated with biologics, with prevalence exceeding 5% of all sJIA patients. Often, progression of LD happens prior to detection; therefore, there is an urgent need to define patients at risk as well as strategies to screen for early onset of this condition (7). Studies have found that sJIA-associated LD may occur due to introduction of IL-1– and IL-6–blocking biologics and a decrease in use of corticosteroids, causing increased drug exposure (7, 8). It is plausible that the prolonged usage of biologics in these patients could adversely impact the progression of LD. Since the timing and role of allo-HSCT in refractory sJIA is yet to be defined, patients experiencing sJIA-associated LD could be considered ineligible candidates for allo-HSCT since the indication for allo-HSCT is defined individually (7, 10).

IFNγ is a key factor in the development of MAS. Gene expression studies of patients treated with the IL-1 receptor antagonist anakinra revealed an increase of IFN-inducible genes (6). Emapalumab is a human IgG1 anti-IFNγ monoclonal antibody that binds to IFNγ, both free and receptor-bound, which inhibits receptor dimerization and transduction of IFNγ signaling and neutralizes its biologic activity (9). Emapalumab has shown to be favorable in dampening hyperinflammation in patients with primary HLH prior to proceeding to allo-HSCT; however, its role in MAS or secondary HLH is yet to be defined (9). Given the key role of IFNγ in driving the underlying inflammation, it is intriguing that emapalumab could be effective in MAS in refractory cases of sJIA prior to allo-HSCT.

Traditionally, autologous HSCT has been studied in children with sJIA. Children undergoing autologous HSCT have demonstrated drug-free remission; however, in some cases children have relapsed with inflammatory symptoms and recurrence of MAS. More recently, allo-HSCT for severe sJIA has been shown to be safe and effective, including in patients who do not respond to intense treatment or who have developed complications associated with MAS (10). Our patient was admitted to the intensive care unit on three separate occasions, with each stay ranging from 2 to 4 days in length. One admission secondary to pneumonia and acute respiratory distress lasted approximately 10 days; during that stay, the patient required intubation and mechanical ventilation. Allo-HSCT was considered a last option for this patient, as she had exhausted all medical treatment and was developing multiple steroid-related toxicities.

This case study demonstrates that achieving remission of MAS using an IFNγ-targeting approach with emapalumab is critical in optimizing the outcome of allo-HSCT for this rare indication. A reduced intensity allo-HSCT is feasible in patients with refractory sJIA complicated by MAS, which could also potentially halt the progression of associated LD. A limitation to this case study is that this is a single case report and extrapolating this data could be challenging. Data on emapalumab for its use in patients with secondary forms of HLH, including MAS, is scarce.

The evidence presented in this case report provides support for further studies evaluating the use of emapalumab for control of MAS in patients with refractory sJIA and associated LD prior to allo-HSCT who are unable to achieve disease control with other available biological agents.

## Data Availability

The original contributions presented in the study are included in the article/Supplementary Material, further inquiries can be directed to the corresponding author.
